# Effect of nonsurgical periodontal treatment in patients with periodontitis and rheumatoid arthritis: A systematic review

**DOI:** 10.4317/medoral.20974

**Published:** 2016-03-06

**Authors:** Francisco-Javier Silvestre, Javier Silvestre-Rangil, Leticia Bagan, Jose V. Bagan

**Affiliations:** 1 Assistant Professor. Department of Stomatology, University of Valencia; 2 Associate Professor. Department of Stomatology, University of Valencia; 3 Associate Professor of Oral Medicine. European University of Valencia; 4 Professor and Chairman of Oral Medicine. Department of Stomatology, University of Valencia

## Abstract

**Background:**

Periodontitis has been regarded as a potential risk factor for rheumatoid arthritis (RA). A systematic review is made to determine whether nonsurgical periodontal treatment in patients with RA offers benefits in terms of the clinical activity and inflammatory markers of the disease.

**Material and Methods:**

A search was made of the Medline-PubMed, Cochrane, Embase and Scopus databases to identify studies on the relationship between the two disease processes, and especially on the effects of nonsurgical treatment in patients of this kind. The search was based on the following keywords: *rheumatoid arthritis AND periodontitis* (MeSH), *rheumatoid arthritis AND periodontal treatment*.

**Results:**

Eight articles on the nonsurgical treatment of patients with periodontitis and RA were finally included in the study. All of them evaluated clinical (DAS28) and laboratory test activity (ESR, CRP, IL-6, TNFα) before and after treatment. A clear decrease in DAS28 score and ESR was recorded, while other parameters such as CRP, IL-6 and TNFα showed a non significant tendency to decrease as a result of treatment.

**Conclusions:**

Nonsurgical treatment improved the periodontal condition of patients with periodontitis and RA, with beneficial effects upon the clinical and laboratory test parameters (DAS28 and ESR), while other inflammatory markers showed a marked tendency to decrease. However, all the studies included in the review involved small samples sizes and follow-up periods of no more than 6 months. Larger and particularly longitudinal studies are therefore needed to more firmly establish possible significant relations between the two disease processes.

**Key words:**Periodontitis, rheumatoid arthritis, periodontal treatment.

## Introduction

Periodontal disease (PD) is one of the most frequent oral disorders, particularly in elderly patients, and is one of the most common causes of tooth loss. Periodontitis causes chronic inflammation, with the constant release of inflammatory mediators, and may be a risk factor for the development of other systemic inflammatory disorders (1).

There is now considerable evidence supporting the association between periodontitis and certain chronic inflammatory processes such as cardiovascular disease, diabetes mellitus, obesity and certain alterations during pregnancy. In recent years rheumatoid arthritis (RA) has also been included among these potentially associated disorders (2).

Rheumatoid arthritis is an autoimmune disease characterized by chronic inflammation of the joints, particularly the small joints of the hands and feet, with progressive destruction resulting in variable degrees of deformity and functional disability. Inflammation plays a key role in the origin of RA, its chronification, and in progression of the disease with soft and hard tissue destruction, in a way similar to the situation seen in patients with chronic periodontitis (3).

Most of the mentioned associations can be explained in part by an excessive secretion of cytokines and other inflammatory mediators produced in response to an altered host immune reaction. There appears to be an imbalance between proinflammatory and antiinflammatory mediators that would be the cause of the tissue damage. Certain cytokines are ultimately correlated to such damage, such as interleukin 1 (IL-1), tumor necrosis factor-α (TNFα) and prostaglandin E2 (4-6).

A number of hypotheses have tried to explain the relationship between PD and RA. The currently most widely accepted hypothesis is the “double hit model”, which postulates that a first “hit” in the form of inflammation due to periodontitis is followed by a second “hit” at joint level, with an exacerbation of the inflammatory response in these locations. Another hypothesis points to the development of an autoimmune response generated by proteins partially altered by enzymes of bacterial origin, such as anti-citrullinated protein antibodies in RA (7-9).

A number of laboratory test parameters (acute phase reactants) may experience clear alterations during the active phases of RA, such as C-reactive protein (CRP), erythrocyte sedimentation rate (ESR), rheumatoid factor (RF), interleukin 1β (IL-1β), interleukin 6 (IL-6) or TNFα (5,10).

With regard to this possible bidirectional relationship between PD and RA, changes have been described in the aforementioned biochemical markers after nonsurgical *periodontal treatment*, implying a decrease in the inflammation generated at periodontal level, and this in turn could exert a positive influence upon RA.

The aim of this study was to conduct a systematic review of the literature supported by a certain level of evidence, in an attempt to determine whether nonsurgical *periodontal treatment* in patients with RA offers benefits in terms of the clinical activity and inflammatory markers of the disease.

## Material and Methods

- Search strategy

Two search steps were established in the present review. In a first step we conducted a search of the Medline-PubMed database to retrieve articles that had studied the possible relationship between PD and RA, focusing particularly on the effect of nonsurgical periodontal treatment in patients with arthritis and periodontitis. The search covered the period up to February 2015, and was limited to articles published in English.

The following descriptors were used: *rheumatoid arthritis AND periodontitis* (MeSH), resulting in a total of 362 references which were then analyzed on the basis of the title of the article or the abstract, and the index terms included in the publication. The following inclusion criteria were applied:

1) Controlled clinical studies on the effect (intervention) of nonsurgical periodontal treatment in patients with RA and periodontitis.

2) Patients diagnosed with RA and periodontitis and aged over 30 years.

3) The absence of systemic inflammatory diseases capable of influencing RA or periodontitis.

4) The absence of antibacterial drug use in the three months before periodontal treatment.

5) The absence of periodontal treatment at least during the previous 6 months.

6) The presence of an age- and gender-matched control group without periodontal treatment.

7) Articles published in English.

In a second step we conducted a search in the Medline-PubMed, Cochrane, Embase and Scopus databases, using the following terms: *rheumatoid arthritis AND periodontal treatment.* A total of 191 articles were identified. After screening both searches according to the established inclusion criteria, and after eliminating duplicate studies, a total of 8 articles were obtained ( Table 1): 7 controlled studies (11-17) and one clinical trial (18). In addition, 31 reviews were retrieved, though only one systematic review and metaanalysis of periodontal treatment in RA was identified (19).

**Table 1:**
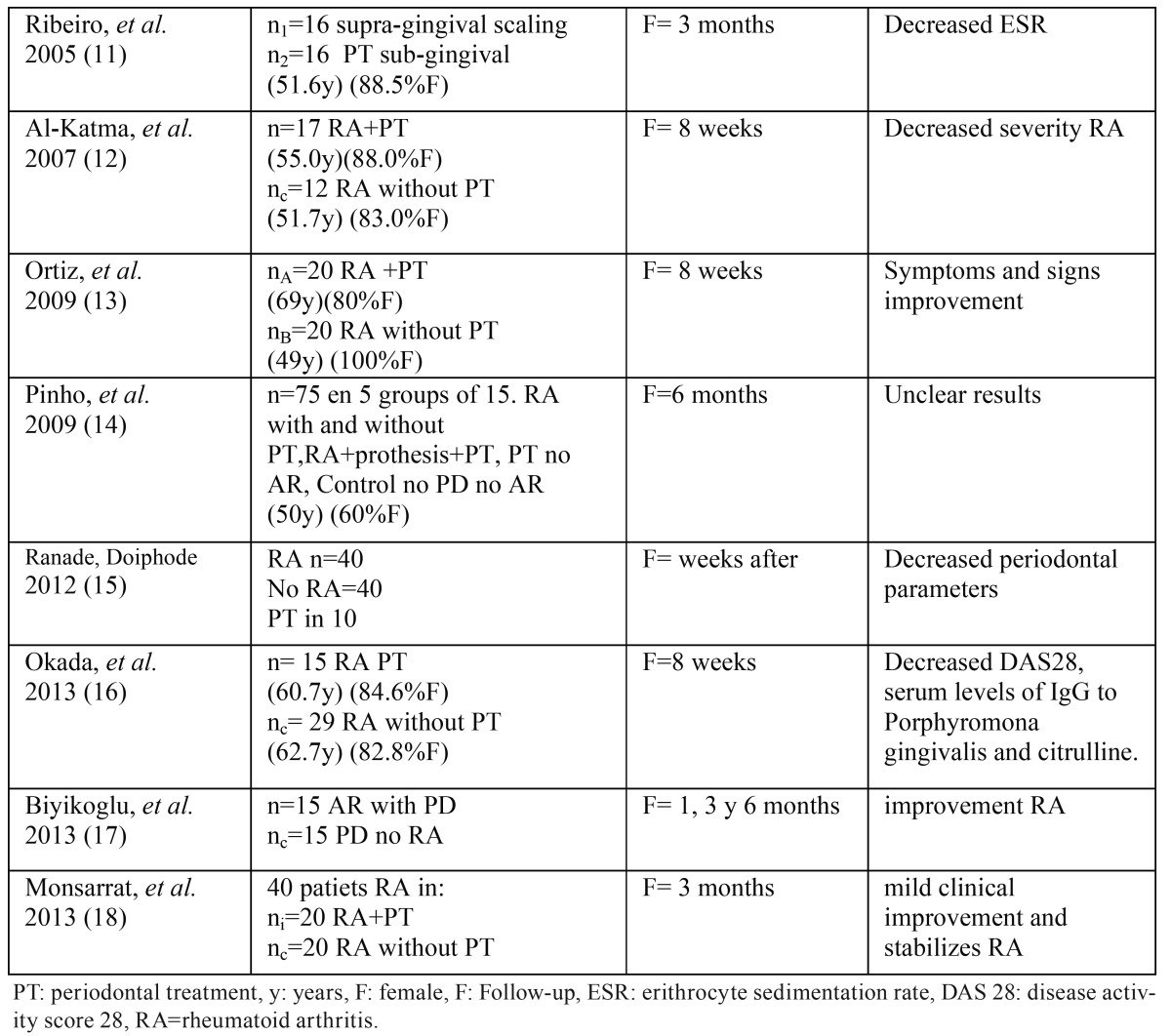
Sample characteristics, monitoring and conclusions of the 8 studies included.

## Results

The 8 studies finally included in the analysis focused on the nonsurgical periodontal treatment of patients with RA and periodontitis. Periodontal treatment included instructions referred to oral hygiene, supragingival (16) and subgingival cleaning with ultrasound, and root rasping and scaling (11-15,17,18). One study administered antibiotic treatment and an antiseptic rinse (18), while the study published by Ranade *et al.* (15) moreover included occlusal adjustment.

The study parameters were assessed at baseline before the intervention and over a follow-up period of 6 weeks (13), 8 weeks (12,16), three months (11,18) and 6 months (14,17). The study of Ranade *et al.* (15) only specified that evaluation was made weeks after periodontal treatment.

In general, all the studies evaluated the changes in the activity of the disease (RA) associated to treatment of the periodontal infection and inflammation, based on the measurement of different clinical and laboratory test parameters. The inclusion criteria also controlled comorbidities with a view to ensuring that there were no other concomitant inflammatory conditions capable of altering the results. In addition to examining the effect of the intervention, Pinho *et al.* (14) established comparisons according to RA treatment with antirheumatic agents or disease-modifying antirheumatic drugs (TNFα inhibitors). The sample size referred to the patients with RA subjected to periodontal treatment varied from 10 (15) to 75 (14).

In 7 studies the evaluation of disease activity was based on the Health Assessment Questionnaire (HAQ) (11,15) and the Disease Activity Score in 28 joints (DAS28). In some cases CRP was measured as acute phase reactant, while others studies recorded ESR. The DAS28 score tended to decrease after non surgical periodontal treatment. Seven studies (85.7%) evidenced clear clinical improvement of RA after treatment (11-13,15-18), and in one case improvement of the periodontal parameters was greater in the RA group than among the non-RA controls.

Both ESR and CRP are systemic inflammatory markers that can be used to determine the degree of activity of the disease (RA) at a given point in time. In this regard, a clear decrease in ESR was observed following periodontal treatment (11-14), though the decrease in CRP failed to reach statistical significance. Likewise, other pro inflammatory cytokines such as TNFα and IL-6 also tended to decrease, though not to a significant extent (19).

Rheumatoid factor (RF) has been used for years as a marker for assessing RA, though it lacks specificity. In fact, 15% of all arthritic patients may prove seronegative for RF. None of the studies evaluated by Kaur *et al.* (19) demonstrated changes in RF following non surgical periodontal treatment.

Anti-citrullinated protein antibodies (ACPAs) are a result of inflammation, and their presence in serum is quite specific of RA (95%). These antibodies were evaluated in the study published by Okada (16), though no significant differences were found.

## Discussion

- Common physiopathological mechanisms

Both rheumatoid arthritis (RA) and periodontal disease (PD) may share common physiopathological mechanisms characterized by chronic inflammation in which the end result is bone reabsorption of the supporting structures or alterations of the small body joints (2). These disorders involve a certain immune susceptibility combined with persistent inflammation which in the case of periodontal disease (PD) is produced by sub gingival bacterial plaque. The periodontal inflammatory state maintains synergic interaction with the plaque to increase its protection against the host defense mechanisms, acquire nutrients, and create an environment favorable to perpetuation of the bacterial plaque. This situation gives rise to a feedback mechanism between the plaque and the tissue inflammation known as “inflammophilia” (20,21).

Rheumatoid arthritis is characterized by different stimuli that can induce an arthrogenic inflammatory reaction with the influence of endogenous products such as certain connective tissue proteins or even altered immunoglobulins. Certain bacteria capable of causing periodontal disease, such as *Porphyromona gingivalis* (Pg), are able to produce the citrullination of host proteins, mediated by peptidyl arginine deiminase (PAD), transforming them into antigens (22).

- Citrullination

Until some time ago, the only marker for monitoring RA was the laboratory test determination of rheumatoid factor (RF), an antibody targeted to the constant fraction (Fc) of IgG. In fact, RF assessment is regarded as a diagnostic criterion for RA. However, RF is neither sensitive nor specific, since it can also be found in other autoimmune disorders, in oncological diseases, chronic infections, and even in normal elderly people (23).

At present, other antibodies have been associated to arthritis, such as antifilaggrin or antiprofilaggrin antibodies, which act against the antiperinuclear factor (APF). Filaggrin is an epidermal protein that establishes bonds between the keratin intermediate filaments during the terminal stages of epithelial cell differentiation, while profilaggrin is the precursor composed of 10-12 filaggrin molecules (24). Mention also must be made of antikeratin antibodies (AKAs) and anti-Sa antibodies in which citrulline is implicated as antigenic determinant, and vimentin. The latter is a fibrous protein that forms intermediate filaments of the cytoskeleton, and is widely expressed in embryonic, endothelial and blood cells. As a proinflammatory agent, tumor necrosis factor-α can induce the secretion of vimentin, while interleukin 10 (IL-10), as an inhibitory cytokine, blocks vimentin (25).

Other intervening antibodies are anti-citrullinated cyclic peptide antibodies (ACCPs), which are very sensitive (80%) and specific (98%) of RA. These antibodies moreover manifest in very early stages of the disease (26).

In general, the proteins implicated as antigenic stimuli in the pathogenesis of RA have suffered post-translation changes following their synthesis – one of the most common modifications being citrullination. The latter involves the conversion of an arginine group into a citrulline group, catalyzed by peptidyl arginine deiminase (PAD) in the presence of calcium. The result is a change in molecular weight and a loss of positive electrical charge, modifying the capacity of the molecule to interact with other proteins and making it more vulnerable to proteolytic degradation mediated by other enzymes (27).

Five PAD isotypes have been identified, each with a specific tissue expression profile. In this regard, PAD 1 is mainly expressed by the epidermis and uterus; PAD 2 is expressed by skeletal muscle, the spleen, brain and salivary glands among other tissues; PAD 3 is expressed by hair follicles; PAD 4 is expressed by neutrophils and eosinophils; and PAD 5 has been detected in ovaries, testicles and peripheral blood leukocytes (28).

The activity of PAD appears to be influenced by a range of estrogenic compounds. It also must be taken into account that during apoptosis, some cell proteins are citrullinated - particularly vimentin. In this respect, citrullination can induce almost complete depolymerization, resulting in alteration of the cytoskeletal network (29).

However, PAD 2 and PAD 4 are the only isotypes acting upon proteins such as fibrin and vimentin in the synovial tissue of patients with arthritis, and can generate an immune response by converting into antigenic elements. Specifically, PAD 4 reportedly may react in the serum of patients with RA, thereby intrinsically acting as a neoantigen (30).

The immune response may be mediated by B cells, amplifying in susceptible individuals and generating plasma cells with the secretion of antibodies. Likewise, T lymphocytes may act against the citrullinated proteins of the synovial membrane, which constitutes the target organ of the immune response.

Clinical studies of early arthritis have identified highly specific antibodies targeted to citrullinated peptides and which might serve as diagnostic markers of the disease. The fibrin alpha- and beta-chains are relevant citrullinated proteins in inflammatory synovial tissue, and the antibodies targeted to these molecules have greater specificity for RA (31,32).

Although the diagnosis of RA is presently fundamented upon patient anamnesis and physical examination, and on the findings of the radiological studies, the evaluation of antibodies against citrullinated proteins may be of great help, particularly in patients with negative RF titers, and when the disease presents a poorer diagnosis (27).

- Periodontal treatment and disease activity

Regarding the possible relationship between periodontal treatment and disease activity, it has been suggested that the control of periodontal disease could contribute to lessen infection and periodontal inflammation through the adoption preventive measures with good oral hygiene, supragingival and sub gingival cleaning with ultrasound, and root rasping and scaling. These measures could reduce the clinical activity of RA, with a decrease in the serum levels of certain products derived from the inflammatory process (13).

Our systematic review indicates that the application of conservative treatments clearly improves the periodontal parameters (bleeding upon probing, pocket depth and attachment loss) in patients with RA and chronic periodontal disease (15). Furthermore, this periodontal improvement was seen to be associated to beneficial effects in relation to other disease assessment parameters such as the DAS28 score (12,13,16) and ESR (11-14). In this regard, ESR decreased significantly – suggesting a reduction in systemic inflammation following non surgical periodontal treatment.

The DAS28 score showed a tendency towards lessened signs and symptoms of disease, with a decrease in the number of painful joints, and lesser morning stiffness and joint effusion, independently of the drugs used to treat RA. Although Pinho *et al.* (14) found the differences between the studied groups to be significant at three months of follow-up, significance was lost after 6 months. The follow-up periods in the different studies ranged from 6 weeks to 6 months, and might be too short to detect clinical changes referred to periodontal infection and inflammation.

Regarding other analyzed laboratory factors, RF was evaluated in three studies, while IL-6 and anti-citrullinated cyclic peptide antibodies (ACCPs) were studied in another. However, other authors have shown that periodontal treatment clearly reduces the CRP concentrations in patients without RA. Few of the studies included in the review analyzed the most important inflammatory parameters; the sample sizes were small; and the fact that the patients were receiving different drugs for arthritis (antiinflammatory drugs and disease-modifying antirheumatic drugs) may have been a confounding factor. Furthermore, in many cases the randomization method used was not clear.

While the existing level of evidence is low, it seems clear that a decrease in periodontal inflammation in some way influences the level of systemic inflammation, and appears to contribute to clinical improvement of the disease.
